# The Use of Micro- and Nanocarriers for Resveratrol Delivery into and across the Skin in Different Skin Diseases—A Literature Review

**DOI:** 10.3390/pharmaceutics13040451

**Published:** 2021-03-26

**Authors:** Beata Szulc-Musioł, Beata Sarecka-Hujar

**Affiliations:** 1 Department of Pharmaceutical Technology, Faculty of Pharmaceutical Sciences in Sosnowiec, Medical University of Silesia, Kasztanowa Str 3, 41-200 Sosnowiec, Poland; 2 Department of Basic Biomedical Science, Faculty of Pharmaceutical Sciences in Sosnowiec, Medical University of Silesia, Kasztanowa Str 3, 41-200 Sosnowiec, Poland; bsarecka-hujar@sum.edu.pl

**Keywords:** resveratrol, skin, transdermal delivery, liposome, microparticle, nanoparticle, nanoformulations, skin disease

## Abstract

In recent years, polyphenols have been extensively studied due to their antioxidant, anticancer, and anti-inflammatory properties. It has been shown that anthocyanins, flavonols, and flavan-3-ols play an important role in the prevention of bacterial infections, as well as vascular or skin diseases. Particularly, resveratrol, as a multi-potent agent, may prevent or mitigate the effects of oxidative stress. As the largest organ of the human body, skin is an extremely desirable target for the possible delivery of active substances. The transdermal route of administration of active compounds shows many advantages, including avoidance of gastrointestinal irritation and the first-pass effect. Moreover, it is non-invasive and can be self-administered. However, this delivery is limited, mainly due to the need to overpassing the stratum corneum, the possible decomposition of the substances in contact with the skin surface or in the deeper layers thereof. In addition, using resveratrol for topical and transdermal delivery faces the problems of its low solubility and poor stability. To overcome this, novel systems of delivery are being developed for the effective transport of resveratrol across the skin. Carriers in the micro and nano size were demonstrated to be more efficient for safe and faster topical and transdermal delivery of active substances. The present review aimed to discuss the role of resveratrol in the treatment of skin abnormalities with a special emphasis on technologies enhancing transdermal delivery of resveratrol.

## 1. Introduction

The skin, as the outermost covering, is the largest organ of the human body. It consists of many layers, including the epidermis, dermis, and subcutaneous tissue. All of these layers are built of cells characterized by specific functions. Such a structure provides an effective barrier and it is the first line of body defense against radiation, pathogens, and excessive water loss [1]. Since skin is the largest organ in humans, new opportunities for drug delivery using this route are sought after. The transdermal route of administration of active substances is however limited, mainly due to the need to overcome the stratum corneum, the possible decomposition of the active substances in contact with the skin surface or in the deeper layers thereof. In addition, irritations, allergic responses, or skin damage associated with prolonged use of some preparations are also observed [2,3]. 

Natural substances which may bring benefits in the treatment of dermatological changes cover a wide panel of those extracted from plant and include polyunsaturated fatty acids, diterpenes and triterpenes, some saponins, sterols, or polyphenols. Polyphenols are one of the most popular classes of natural compounds and contain various subclasses, i.e. simple phenols, phenolic acids, stilbenes, and favonoids (flavanones, flavones, dihydroflavonols, flavonols, flavan-3-ols, anthocyanidins, and proanthocyanidins) [4]. However, numerous bioactive substances are unstable when isolated from a natural source and therefore must be protected from the external environment. Regarding formulations for topical use (i.e., cosmetic or dermatologic), their main goal should be the appropriate active substance concentration in the skin tissue (skin retention) which may be difficult due to the specific skin structure. That is, the non-polar environment of the stratum corneum is a barrier through which many active substances, even the lipophilic ones, penetrate with difficulty, without reaching the deeper layers of the skin and thus not causing the assumed biological effect [5]. Moreover, the vital epidermis is also considered a significant barrier of permeability [6].

In recent years, extensive studies have been performed on the use of polyphenols in therapy for various disorders due to their antioxidant, anticancer, and anti-inflammatory properties [5,7,8]. Anthocyanins, flavonols, and flavan-3-ols play an important role in the prevention of bacterial infections, especially urinary tract infections (UTI) [9,10,11]. A promising strategy to prevent or mitigate the effects of oxidative stress may be the result of topical administration of antioxidants, in particular resveratrol [12]. Since skin permeation of an active substance is determined by its properties, lipophilic molecules with low molecular weight may penetrate the stratum corneum more readily [13]. Similarly, it is important whether the compound is in neutral or ionic condition. Resveratrol in neutral condition was shown to have higher permeability than resveratrol in the ionized condition [14,15].

The aim of the present study was to discuss the role of resveratrol in treatment of skin abnormalities with a special emphasis on technologies enhancing transdermal delivery of resveratrol.

## 2. Biological Activity of Resveratrol

Resveratrol (3,5,4′-trihydroxy-trans-stilbene) is a natural phenolic compound, occurring in a high concentration in the root of the knotweed (*Polygonum cuspidatum*) grown in Japan and China for the purposes of local folk medicine [16]. The Kojo-kon preparation is produced from the extract of knotweed, used to treat purulent skin infections, mycoses, and venereal diseases [17]. Different specimens of vines, peanuts, and berries are also rich in resveratrol [18]. It is a naturally occurring phytoalexin produced in plants during the stress of attack by pathogens [19].

Resveratrol shows low aqueous solubility as well as poor chemical stability. It exists in two isomeric forms, trans-resveratrol and cis-resveratrol. The trans form is considered to be biologically active and of higher stability [20]. The cis isomer has been reported to lack any pharmacological activity owing to rapid glucuronidation, which results in a lower bioavailability and effectiveness [21]. In solution, trans-resveratrol isomerized to the cis form. It is difficult to maintain bioefficacious levels of resveratrol in the human blood and in target tissues since it shows rapid clearance from the circulation. It was demonstrated that in plasma, only unchanged resveratrol in a concentration below 5 ng/mL could be detected [22]. The limitations in bioavailability of resveratrol were suggested to be related to an extremely rapid sulfate conjugation by the intestine/liver [22]. The unfavorable features of resveratrol include among others: susceptibility to oxidation, photosensitivity, and cytotoxic effect in higher total dosages, though relatively high local concentrations are required for an effect [23,24].

Resveratrol has many different healing and preventive properties, including antioxidant, anti-inflammatory, antimicrobial, chemopreventive, and immunomodulatory effects [25,26,27,28,29,30]. It may affect the activity of the nicotine adenine dinucleotide (NAD)+-dependent histone/protein deacetylases (sirtuins) involved in modulating numerous cellular pathways which are related to stress responses and aging, e.g. regulation of p53, fatty acid metabolism, or cell cycle regulation [31,32]. Sirt1 is a key modulator of pathways associated with inherited dermatologic diseases and skin cancers [33].

Numerous preclinical and clinical studies have confirmed the efficacy of this phytoalexin in the treatment of diabetes [30,31,32], neurodegenerative diseases [34,35], cancer [36,37,38], aging [39], obesity [40,41], cardiovascular disease [42,43], and regeneration of bone tissues [44]. Resveratrol, as a compound of natural origin with proven anti-inflammatory and antimicrobial activity, seems to be an alternative to synthetic drugs used in vaginal therapy, especially with the observed spread of antimicrobial resistance, mainly in *Neisseria gonorrhoeae* [45]. This natural plant compound was found to significantly reduce alveolar bone loss and attenuate oxidative stress during experimental periodontitis [46]. Resveratrol is used in functional foods for skin care and health [47,48,49,50].

## 3. Resveratrol in Skin Diseases: In Vitro and In Vivo Studies

In skin diseases, topical treatment reduces side effects and can provide high drug levels at the site of the disease when compared to oral treatment. A promising strategy in preventing or alleviating the effects of oxidative stress may be the administration of topical antioxidants, including resveratrol. Since resveratrol is a potent antioxidant agent, it may be used for the effective management of different skin conditions like extrinsic skin aging, psoriasis, acne vulgaris, irritant contact dermatitis (ICD), and vitiligo [51,52,53].

### 3.1. Resveratrol as an Antimicrobial Agent

In recent years, resveratrol has been extensively studied for its effects on human pathogens. Many studies have shown the antimicrobial activity of resveratrol against a wide range of bacteria, viruses, and fungi [54]. Interestingly, it was demonstrated that resveratrol is able to inhibit replication of human herpes viruses [55], influenza viruses [56], and Epstein–Barr virus [57,58]. Resveratrol was demonstrated to significantly inhibit Middle East respiratory syndrome coronavirus (MERS-CoV) infection as well as prolong cellular survival after virus infection [59,60]. The study by Ferreira et al. [61] analyzed the effect of resveratrol against Leishmania amazonensis and showed both antipromastigote and antiamastigote effects of resveratrol used alone or in combination with amphotericin B. For the combination of the compounds, synergy action for L. amazonensis was also found [61].

Natural antimicrobials seem to hold promise as an alternative to antibiotics and can be used to decrease the spread of resistant strains. A few studies have investigated the therapeutic application of resveratrol against infectious diseases in animal models or human trials.

Acne vulgaris is one of the most common dermatological diseases. It is estimated that about 94–95% of the pubertal population and 20–40% of adults suffer from acne [62]. It is characterized by the overproduction of corneocytes causing obstruction of the sebaceous follicles, androgenic stimulation of the sebaceous glands, and the presence of bacteria from the Propionibacterium acne family. Negative feedback or excessive stimulation of the inflammatory process, resulting in increased function of the sebaceous glands, were suggested to cause the occurrence of accompanying clinical symptoms [63]. In vitro studies showed that resveratrol revealed sustained antibacterial activity against Propionibacterium acnes [64]. Docherty et al. [65] observed that the highest concentration of resveratrol tested (200 mg/L) was bactericidal, whereas lower concentrations (50 and 100 mg/L) were bacteriostatic. Topical application of phenolic compounds (20 μg resveratrol, quercetin, gallic acid) reduced the inflammatory process and oxidative stress induced by Propionibacterium acnes in mice with ear edema [66].

A clinical trial published by Fabbrocini et al. [67] showed that a topical formulation containing 0.001% trans-resveratrol was effective in reducing the severity of acne vulgaris. In a single blind study, twenty subjects with facial acute vulgaris were treated for 60 days with a hydrogel containing resveratrol. Acne seriousness was assessed using two grades: the Global Acne Grading System (GAGS) and a skin biopsy. After the end of treatment, a 53.75% and 6.1% reduction in GAGS score was observed for the test and control groups, respectively, compared to baseline. Furthermore, the histologic evaluation of cyanoacrylate follicular biopsies showed a decrease in the total area by 66.7% and in the density of microcomedones by 9.7% on the preparation-treated side of the face, compared to baseline [67].

The herpes simplex virus (HSV) is one of the most prevalent human pathogens. Herpes simplex virus type 1 (HSV-1) is associated mainly with oral and facial infections, whereas herpes simplex virus type 2 (HSV-2) infection involves genital, perigenital, or anal skin sites [68]. The primary infection of the mucosa causes ulceration and blistering, and subsequently enters sensory neurons. Next, it reaches the dorsal root ganglia, where it causes latent, lifelong infection [69]. During reactivation, the virus is transported to the primary infection site, i.e. to the mucosal tissues [70].

Docherty and colleagues [71] observed that resveratrol inhibited both HSV-1 and HSV-2 replication, in monkey-kidney (Vero) and in human-lung cell lines (MRC-5), infected with HSV-1 and HSV-2, in a dose- and time-dependent manner. At the dose of 25 µg/mL of resveratrol, approximately 95% inhibition of viral replication was observed, while at 50 µg/mL, replication was almost completely abrogated by 72 h. Further, viral replication was inhibited as long as resveratrol was present, its removal resulted in a resumption of HSV replication. According to the authors, resveratrol affected viral replication by reducing the expression of immediate–early viral protein-4 (ICP-4) [71]. In another study [72], the authors observed that applying a cream containing resveratrol at a concentration of 12.5 and 25% five times a day to skin lesions in SKH1 hairless mice infected with HSV-1 was more effective in inhibiting lesions compared to the control group. Similar to in vitro studies [71], the efficacy of resveratrol in reducing skin lesions was found to be influenced by the concentration of resveratrol, the timing of treatment initiation, and the number of applications per day. In addition, a comparative study showed that resveratrol exerts a lesion suppression effect comparable to acyclovir and superior to docosanol [72]. The same research group [73] also conducted an in vivo study to investigate the effect of resveratrol on the reduction of vaginal and extra-vaginal lesion formation in mice infected with both HSV-1 and -2. Resveratrol was applied as a cream at a concentration of 6.25%, 12.5%, and 19%. It was shown that vaginal administration of resveratrol significantly inhibited viral replication compared to the placebo; in addition, 19% resveratrol cream showed efficacy comparable to 5% acyclovir ointment. Mortality rates were demonstrated as follows: 37% in placebo-treated animals, 40% in animals treated with 6.25% resveratrol, 24% in animals treated with 12.5% resveratrol, and 3% in animals treated with 19% resveratrol [73]. Thus, with an increasing amount of resveratrol, decreased mortality rates in the studied animals were observed. Similarly, positive results were obtained for the resveratrol derivative oxyresveratrol (trans-2,4,3′,5′-tetrahydroxystilbene) in HSV-1 infected mice [74]. The authors found that topical application of 15% and 30% oxyresveratrol ointment, five times daily for seven days, significantly reduced lesion development (*p* = 0.03 and *p* < 0.0001, respectively) compared with control. Moreover, it was shown that 30% oxyresveratrol ointment had a lesion suppression effect comparable to 5% acyclovir cream [74].

Resveratrol may inhibit the expression of transcriptional regulatory genes such as saeR, saeS, and hla which may reduce the hemolytic capacity of Staphylococcus aureus [75]. The decreased virulence of S. aureus due to resveratrol was confirmed in mouse abscess model. The drug resistance of planktonic, biofilm, and intracellular bacteria highlights the need to develop new antibacterial agents. Yang et al. [76] investigated the antibacterial activity of pterostilbene, a methoxylated resveratrol derivative, against drug-resistant Staphylococcus aureus as well as for its in vivo capability in skin-infection inhibition. The minimum inhibitory concentration assay showed that pterostilbene exhibited more biocidal activity against Gram-positive bacteria than resveratrol. In addition, the in vitro cytotoxicity test in mammalian cells as well as an in vivo skin irritation test confirmed the safety of this compound.

In turn, Comotto et al. [77] manufactured alginate-based hydrogels with embedded natural antioxidant compounds (curcumin and t-resveratrol). Hydrogels exhibited no toxicity to human keratinocytes (curcumin up to 150 µg/mL and t-resveratrol up to 300 µg/mL). However, curcumin-based alginate dressings were found to be more effective in bacteriostatic activity than resveratrol dressings [77].

### 3.2. Resveratrol in Wound Healing

Chronic wounds are wounds that are difficult to heal and last longer than 6–8 weeks. They include diabetic lesions, bedsores, burns, and acute injuries. Complications in chronic wounds can result from the presence of microorganisms. Natural antioxidants are increasingly used in the treatment of chronic wounds. They not only reduce the concentration of oxygen free radicals, but also stimulate the process of angiogenesis, reduce the inflammatory reaction, promote the proliferation of fibroblasts and keratinocytes, and have antimicrobial properties against microorganisms resistant to the used chemotherapeutics [78].

It seems that resveratrol may play a role in treating cutaneous wound defects in both young and aged individuals, or act as a useful supplement to current treatments such as glucocorticoids [79] and epidermal growth factor (EGF) [80]. It was discovered that chronic topical administration of resveratrol and metformin (MET), but not rapamycin (RAPA), accelerated wound healing with improved epidermis, hair follicles, and collagen deposition in young rodents, and MET exhibited more profound effects [81]. The critical point for cutaneous wound healing is considered to be the moment when blood vessel formation (vasculature formed) in granulation tissues begins [82]. It was shown that locally applied resveratrol and MET improved vascularization of the wound beds, in both young and aged rats [81].

In vivo, the regenerative potential of a biodegradable dressing synthesized from chitosan and sodium hyaluronate with the addition of resveratrol was evaluated on a murine model of disruption of the cutaneous tissue [83]. On the tenth day of the study, it was observed that the wound treated with the sponge had a mean diameter of 2.76 ± 0.4273 mm^2^ compared to the group control which had a mean area of 6.08 ± 0.3889 mm^2^. The results obtained demonstrated that the dressing accelerated the formation of granulation tissue, facilitating wound healing [83].

In another study based on animal model study (Sprague–Dawley rats), Meng et al. [84] investigated the possibility of using bacterial cellulose (BC) combined with resveratrol in skin regenerative medicine and wound healing. BC increased the absorption of exudate, which in turn increased fibrous coagulability and the absorption of necrotic tissue. The porous, dense structure of BC mimics the extracellular matrix (ECM) of the skin and promotes tissue regeneration [85,86]. In the in vitro study, the authors found that resveratrol was released from BC after approximately 10 min, while in turn, in a biocompatibility study on human fat stem cell lines (hASC), adding resveratrol to the BC significantly increased cell growth compared to BC scaffolds without resveratrol. Moreover, immunostaining showed that the BC with resveratrol scaffold promoted both epidermal tissue regeneration and re-epithelialization during wound repair in damaged rat epidermis [84]. Additionally, the BC/resveratrol scaffold regulated epithelial biomarkers to a greater extent than the BC scaffold itself. These results indicated that the BC combined resveratrol created a biocompatible environment for the attachment and growth of stem cells and promoted epithelial regeneration during wound healing.

A study concerning Melinjo seed extract (MSE) demonstrated that it is a rich source of various stilbenoids, including trans-resveratrol as minor constituent [87]. Daily oral administration to mice of an experimental diet containing resveratrol (0.04% *w*/*w*) or MSE (0.1% or 0.5% *w*/*w*) for 12 weeks was shown to reverse the skin thinning process associated with increased oxidative damage in Sod1 mice (−/−) [88]. MSE and resveratrol also normalized the gene expression of Col1a1 and upregulated the gene expression of Sirt1 in skin tissues. Furthermore, the skin disc culture treated with 10  μM resveratrol for 72 h showed a significant increase in the number of outgrowth fibroblasts of Sod1 −/− mice. Flow cytometry analysis indicated that resveratrol treatment significantly decreased intracellular ROS generation in Sod1 −/− fibroblasts. Thus, Watanabe et al. [88] suggested that MSE and resveratrol can be considered safe antioxidants used to delay skin aging in humans.

Keloids are overgrown fibrous connective tissue usually formed at the site of previous skin damage. They are characterized by a particularly large accumulation of mainly type I collagen in the extracellular matrix of the dermis as well as the proliferation of fibroblasts during the wound healing process. Currently, there is no effective pharmacological treatment of keloids, and the mechanisms of their formation are not yet clear [89]. Several studies have demonstrated resveratrol as an antifibrogenic agent [90,91]. The results of the study by Ikeda and co-workers [92] revealed the expression of type I collagen, α-smooth muscle actin, and heat shock protein 47 in resveratrol-treated keloid fibroblasts in a dose-dependent manner. The authors suggested that resveratrol might reduce collagen accumulation by inhibiting TGF-β1 production or through decreased HSP47 expression. Finally, they reported that even at the highest concentration of 100 μM resveratrol, no toxic effects were found on normal skin fibroblasts from a patient who does not have keloid.

### 3.3. The Role of Resveratrol in Melanoma

Melanoma is an aggressive skin cancer with poor responses to therapy and with increasing incidence worldwide [93,94]. Resveratrol shows anti-cancer properties since it can induce apoptosis in tumor cells. In addition, it can prevent metastasis as an anti-angiogenic agent. It was suggested that the anti-tumor activity of resveratrol in melanoma, e.g., growth inhibition, proliferation, and apoptosis of the cancer cells may be mediated by several signaling pathways [95,96]. Previously, resveratrol was demonstrated in vitro and in cultured muscle cell lines as a natural inhibitor of class IA phosphoinositide 3-kinase, which is a key mediator of insulin-like pathways [97]. The downregulation of the insulin-like pathway promotes longevity and is a goal in the treatment of cancer.

The study by Gong et al. [98] showed that resveratrol inhibited melanoma cell viability in dose-dependent manner with 100 µM of resveratrol as the most effective concentration. The authors observed also that resveratrol significantly increased expression of cleaved caspase-9 protein in the human melanoma cells. Additionally, resveratrol influenced the migration and invasion counteracting melanoma progression as well as possibly promoting autophagy by increasing the expression of, e.g., Beclin 1, which was observed in murine melanoma cells [98].

A previous study reported also that resveratrol may enhance radiation sensitivity in melanoma cells [99]. The authors treated human SK-Mel-5 melanoma cells with resveratrol at variable concentrations for 24 h, and next the radiotherapy (XRT) was applied. In the case of using both resveratrol and XRT, the number of colonies of SK-Mel-5 decreased significantly to 35%, 26%, and 12%, depending to the dose of XRT (1 Gy, 2 Gy, and 4 Gy, respectively) compared to using resveratrol alone (decrease to 56%) or XRT alone (decrease to 94% for 1 Gy, 84% for 2 Gy, and 84% for 4 Gy) [99]. This may suggest a synergistic effect of resveratrol and XRT in inhibiting melanoma cells proliferation.

In turn, the study by Cosco et al. [100] showed that the co-encapsulation of resveratrol and 5-fluorouracil in ultradeformable liposomes improved their anticancer activity on human skin cancer when compared to both the free drug form or either the active substance alone. A study using resveratrol-loaded nanocapsules on a melanoma mice model demonstrated reduced cell viability and a decrease in tumor volume compared to free resveratrol [101]. The obtained nanocapsules were of suitable size (below 150 nm), negative charge, and high encapsulation efficiency.

In the study by Niels et al. [102], the skin of tumor-bearing athymic mice showed a significantly lower resveratrol distribution 5 min after a bolus of 75 mg/kg than skin in nontumor bearing athymic mice (9.00 ± 1.00 nmol/g vs. 21.15 ± 7.22 nmol/g). Table 1 presents the summary of effects of resveratrol in skin diseases [67,71,72,74,81,83,84,88,92,98,99,101].

## 4. Micro- and Nanoformulations and Delivery Systems for Topical Use of Resveratrol

Resveratrol is one of the potent lipophilic antioxidants. It belongs to a group of substances which are generally recognized as safe (GRAS) [103]. Due to its low solubility and poor chemical stability, the most advantageous method for its optimal therapeutic activity is to develop a delivery system which will improve these features of resveratrol without modifying it chemically.

Nanomedicine delivery systems are of great and growing interest in medicine, cosmetology, and pharmacy. The encapsulation of the active substance allows to increase their delivery, improves the stability of unstable bioactive compounds, and protects them from unwanted interactions with the environment. Such forms can also be used in the targeted delivery of a bioactive compound to a desired tissue in the body [104]. There are many ways to design encapsulated formulations, and they use a number of excipients during the process, including surfactants, lipids, biopolymers, or mixtures of these ingredients. The encapsulation of active substances in specific carriers may resolve the problem of percutaneous penetration [105].

### 4.1. Microparticles in Topical Delivery of Resveratrol

Eroğlu et al. [106] obtained, by spray drying, biodegradable resveratrol-loaded microparticles consisting of dipalmitoylphosphatidylcholine (DPPC) and hyaluronic acid (HA) for wound healing. The results of cytotoxicity and proliferation studies, as well as oxidative stress parameters obtained from human skin fibroblast cultures, showed that the components of the matrix of microparticles had a synergic effect on the proliferation of human skin fibroblast cells and the presence of resveratrol decreased oxidation in cells. In subsequent studies, the authors impregnated the developed collagen-laminin dermal matrix with resveratrol-loaded HA-DPPC microparticles in order to obtain a synergistic effect on in vivo wound healing in diabetic rats [107]. The results showed that the bioactivity of the prepared formulation (resveratrol loaded microparticle impregnated dermal matrix; DM-MP-RSV) using skin components (collagen, hyaluronic acid (HA), and laminin) enhances the tissue reconstruction process. In the group of animals with applied DM-MP-RSV, the wound was completely healed at the end of the 14th day after its induction. After that time, in animals treated with resveratrol solution or dermal matrix, the wounds were still open. Another study by this research team demonstrated the utility of the designed preparation containing resveratrol microparticles (named Dermalix) in two groups of patients. The first group had a diagnosis of Wagner 1 and 2 grade diabetic foot ulcer, without an infection, while the second group was free of osteomyelitis or necrotizing soft tissue infection [108]. Randomly selected patients received either standard wound care (SWC), which consisted of irrigation and cleaning with sterile saline solution, or SWC + Dermalix for 4 weeks. This was followed by a 2-month follow-up without treatment. It was observed that at the end of the 4-week period, the percentage closures of wounds were determined as 57.82% for Dermalix and 26.63% for SWC groups. The preparation proved to be more effective particularly in the inflammatory and proliferative phases of wound healing. Based on the results of the study, the authors suggested the possibility of using Dermalix as a primary dressing for non-infected diabetic foot wounds.

A level of skin penetration by resveratrol from chitosan-coated and non-chitosan-coated microcapsules was performed in vivo on human volunteers using the non-invasive tape stripping technique by Scalia et al. [109]. The obtained chitosan-coated lipid microparticles were incorporated into a cream (oil-in-water emulsions), mimicking the actual application conditions. The results described in the investigation show that the cream containing chitosan-coated microparticles significantly enhanced the penetration of resveratrol into the human stratum corneum, compared to uncoated lipid microparticles. The increased accumulation in the stratum corneum is important as this is the main site of its action. In addition, the stratum corneum is most vulnerable to environmental oxidative damage. The improved skin absorption induced by chitosan is attributed to its properties such as bioadhesion and reversible opening of epithelial tight junctions [110,111]. Chitosan adheres to the skin surface due to ionic interactions between its positively charged amino groups and the negative charge in the upper cutaneous layers. In this way, chitosan increases the contact of lipid microparticles with the stratum corneum, which facilitates delivery of the encapsulated drug through the skin. In another study, the authors [112] developed lipid microparticles (LM) loaded with trans-resveratrol to increase its photostability in topical preparations. In vitro, using the DPPH assay, the free radical scavenging activity of cream formulations containing free or microencapsulated resveratrol was estimated, while in vivo, its anti-inflammatory effect on methyl nicotinate-induced erythema in the volar forearm of human volunteers was evaluated. The results obtained from this study indicated that the encapsulation of resveratrol in the LMs did not alter its anti-inflammatory activity and did not influence the antioxidant properties in the cream formulation, however significantly decreased the photolability of the polyphenol.

Ravikumar et al. [113] synthetized a resveratrol-loaded solid-lipid microparticle (SLM) gel for chemoprevention of melanoma. In vitro studies proved that resveratrol-loaded SLM gel exhibited antioxidant and tyrosinase inhibitory activities, while in vivo it had a strong antimelanoma effect against B16F10 melanoma cells. Topical application of the microformulations for a period of 15 days, after a period of induction of tumor formation on mouse skin, showed significant tumor reduction.

To counteract skin infections caused by pathogen bacterial strains (i.e., *Staphylococcus aureus* and *Propionibacterium acnes*), lipid microparticles containing resveratrol and R-(+)-limonene have been developed [114]. It has been shown that encapsulating resveratrol in an amorphous state in microspheres prolongs its half-life and avoids inactivation due to isomerization phenomena. Antimicrobial tests against *Staphylococcus aureus* have shown that the empty microspheres themselves possess antimicrobial activity, which is enhanced by the presence of limonene. In contrast, the antimicrobial activity of microspheres loaded with resveratrol was as high as that of empty microspheres, which may be due to the low amount of resveratrol loaded into microparticles. Table 2 summarizes the results of the studies analyzing resveratrol-loaded microformulations for topical application.

### 4.2. Nanoparticles in Topical Delivery of Resveratrol

#### 4.2.1. Lipid Nanoparticles

Lipid nanocarriers exhibit occlusal properties that may be related to their ability to improve skin hydration. Increasing skin hydration could be useful both in treating skin disorders associated with water loss and enhancing penetration of the active substance through the skin [115,116]. Montenegro et al. [117] assessed the relationship between in vitro occlusion factor and in vivo skin hydration for three types of lipid nanocarriers: nanoemulsions (NEs), solid lipid nanoparticles (SLNs), and nanostructured lipid carriers (NLCs). All nanocarriers were prepared using the phase inversion temperature (PIT) method. These lipid nanocarriers contained 1% *w*/*w* resveratrol, and they were prepared using a solid lipid cetyl palmitate, and as liquid lipid—isopropyl myristate. The results indicate that for lipid nanocarriers of a similar size (26–36 nm), not only the crystallinity of the lipid matrix but also the content of solid lipid can significantly affect their occlusive properties. In the clinical study, twelve healthy female volunteers (with average age of 50 ± 3 years) topically administered gels containing lipid nanoparticles within one week. The authors observed a significant increase in skin hydration in the following order of the used nanoparticles: SLNs > NLCs > NEs. In addition, in vitro occlusion effect showed a linear relationship with in vivo skin hydration. On the other hand, no differences in skin hydration were demonstrated when gels containing unloaded and resveratrol-loaded lipid nanocarriers were compared. This may suggest that encapsulation of resveratrol in nanoparticles does not improve skin hydration.

Resveratrol-loaded solid lipid nanoparticles (RSV-SLN) were investigated for their potential use in the prevention and treatment of irritant contact dermatitis (ICD) [51]. Using the probe technique with Precirol ATO 5 as lipid and Tween 20 as surfactant, solid lipid nanoparticles with particle size less than 100 nm were prepared and then incorporated into Carbopol gel [51]. An ex vivo drug deposition study in human skin showed that after 24 h, 77.24 ± 0.26% of the drug remained in the skin from the resveratrol SLN gel and 21.68 ± 0.31% from the plain resveratrol gel. The prepared resveratrol-SLN gel significantly suppressed swelling of the ear and decreased skin water content in BALB/c mice in a dinitrochlorobenzene (DNCB)-induced ICD model, compared to resveratrol plain gel. In addition, the efficacy of the prepared solid lipid nanoparticle-engrossed gel was demonstrated to be equivalent to that of the marketed (containing a corticosteroid) gel [51].

In another study, formulations based on Pickering emulsions stabilized by chitosan/gum Arabic (CH/GA) nanoparticles (with a weight ratio of 1:1) were obtained as vehicles for trans-resveratrol topical delivery [118]. The nanoparticle content of the formulations was 0.5% and 1.5% *w*/*v*. The developed CH/GA nanoparticles had an average size of 109 nm and the polydispersity index (PDI) was 0.218. Skin absorption was investigated ex vivo using Franz diffusion cells and pig skin. Pickering emulsions have shown higher cutaneous retention and lower permeation of resveratrol, in comparison with a control solution based on a 20% *v*/*v* ethanol. The photostability tests showed that after 4 h of UV exposure in Pickering emulsion formulations, the resveratrol content remained unchanged, whereas in the control solution its amount decreased by 68.15%. This protection from photodegradation was mainly attributed to polymeric nanoparticles, which provide a protective effect and a physical barrier against destructive UV radiation through surrounding emulsion droplets [118].

Nanoparticles were also shown to offer several additional advantages such as enhanced tumor accumulation, and improved therapeutic effectiveness. Research has shown an improvement in the capture and accumulation of resveratrol from solid lipid nanoparticles in cancer tissue due to its physiological features, such as disorders and dysfunctions of cancer vessels, which allow solid lipid nanoparticles (SLN) to pass easily through the tumor [119]. In addition, the maintenance of a high concentration of SLN in the tumor for a long time is possible due to the low venous return and lymphatic drainage [120,121].

Rigon et al. [122] produced solid lipid nanoparticles (SLNs), using a sonication method, with an average diameter lower than 200 nm. Developed preparations showed greater tyrosinase inhibitory activity than resveratrol solution. The SLNs containing resveratrol also demonstrated a capacity to inhibit tyrosinase activity, greater than or equal to that of kojic acid. In addition, it has been shown, that the formulation with soy phosphatidylcholine revealed lower amounts of permeated resveratrol than the formulation without soy phosphatidylcholine. Similar results were observed by other authors [123,124,125], demonstrating that higher concentration of soy phosphatidylcholine promotes a decrease in the amount of drug permeation and increases drug cutaneous retention. Phospholipids, such as soy phosphatidylcholine, can form an additional lipid barrier under the skin surface that decreases the drug flux and at the same time promotes an increase in drug deposition in the skin [126].

Chen et al. [127] investigated the potential of solid lipid nanoparticles (SLN) and nanostructured lipid carriers (NLC) for both dermal and transdermal drug delivery. The actives that were studied were epigallocatechin gallate (EGCG), resveratrol, and vitamin E (VE), including VE and VE acetate. The developed formulations demonstrated high uniformity and stability. Both resveratrol and VE lipid nanoparticles provided effective protection of actives against UV-induced degradation. However, lipid nanoparticles did not protect EGCG from UV degradation. The release study of resveratrol lipid nanoparticles using a dissolution system exhibited a sustained release of drug over 70% after 24 h. Lipid nanoparticles significantly enhanced the skin penetration of resveratrol through the stratum corneum.

#### 4.2.2. Liposomes

Since encapsulation is used to improve unsatisfactory bioavailability and stability of active pharmaceutical ingredient (API) and resveratrol is weakly water soluble, liposomes seem to be an ideal solution for this problem. Liposomes are suggested to be non-toxic and have potential to cross the stratum corneum which is poorly permeable to many substances applied to the skin [128]. The structure of liposomes (hydrophilic core and outer phospholipid bilayer) is similar to the structure of biological membranes. The size of the liposomes ranges from a few nanometers to a few micrometers. Those liposomes with a size below 200 nm belong to the nano-structures and are called nanoliposomes [129]. In the case of transdermal transport of a substance encapsulated in liposomes, the liposome membrane is easily fused to the cell membrane, which greatly facilitates this penetration. Liposomes protect the API against the influence of adverse environmental conditions or against enzymatic degradation. They can also release active substances only when they reach a targeted drug delivery in the body. Data indicate that possible modifications of the composition of liposomes significantly improve their percutaneous penetration [130]. They can also liquefy and, in turn, penetration of the active substance in contact with the membranes of epidermal cells is easier [131]. Previously, it was demonstrated that the maximum fluorescence in the skin was observed for liposomes having 71 nm diameter and the liposomes with diameter of 120 nm showed statistically better penetration of carboxyfluorescein into the skin than those with larger size [132].

The findings of clinical trials presented in the Kwon and co-workers study pointed out that co-encapsulation of resveratrol and 4-n-butylresorcinol (4nBR) in liposome may be a potential therapeutic applications approach against the treatment of melasma [133]. Patients applied cream containing liposome-encapsulated 4nBR and RSV for 4 weeks. Clinical assessments showed that the synergistic action of 4nBR and RSV contributed to the rapid onset of depigmentation after only 2 weeks.

Bonechi and co-workers [134] obtained zwitterionic (DPPC/CHOL) and cationic (DPPC/DC-CHOL) liposomes. The zwitterionic liposomal formulation (DPPC/CHOL (zwitterionic) consisted of DPPC (1,2-dipalmitoyl-sn-glycero-3-phosphocholin) and cholesterol at two different molar ratios: 50/50 and 75/25. A second liposomal formulation, DPPC/DC-CHOL(cationic), contained DPPC and DC-Chol (3ß-[N-(N′,N′-dimethylaminoethane)-carbamoyl]cholesterol) at the same molar ratios, DPPC/CHOL and DPPC/DC-CHOL liposomes. Characterization of the plain and resveratrol-loaded liposomes was conducted in terms of their size, surface charge, and structural details, as well as their molecular scale. None of the investigated systems showed a marked toxicity level toward mouse tumoral fibroblasts NIH3T3 and human astrocytes U3763-MG as evidenced by viability data obtained from neutral red capture and optical microscopy analysis.

Doppalapudi et al. [52] demonstrated that the combination of psoralen and resveratrol may act as a promising therapeutic strategy in vitiligo, which is a chronic skin disease involving its depigmentation due to their dual mechanism of action via stimulating integumental pigmentation and reduction of oxidative stress. Therefore, they formulated ultra-deformable liposomes (UDLs) together loaded with psoralen and resveratrol, and evaluated their efficacy using the B16F10 cell line. Findings from this study revealed that co-loaded liposomes showed significant stimulation of melanin and tyrosinase activity, while in vitro antioxidant assays (ABTS and DPPH assay) exhibited potential free radical scavenging activity. In vitro studies and kinetic showed that the carrier can sustain the release of both drugs over prolonged periods of the time.

Another group [135] prepared liposomes co-loaded with resveratrol and gallic acid and modified those carriers with co-solvent. The penetration enhancer-containing vesicles (PEVs) were obtained by adding propylene glycol as co-solvent while the glycerosomes, by adding glycerol [135]. Resveratrol and gallic acid were selected as curative agents due to their antioxidant, and antimicrobial activity [136,137,138,139,140]. It has been shown that the presence of propylene glycol or glycerol increased the viscosity of the vesicle formulations, positively affecting their stability. In vitro skin penetration and permeation studies demonstrated that accumulation of hydrophilic gallic acid in the skin was 3–4.5 times greater from vesicle systems, especially glycerosomes, than that obtained from dispersion. In addition, there was low toxicity and significant ability of vesicle formulations to protect the keratinocytes HaCaT and fibroblasts 3T3 cells against hydrogen peroxide-induced stress. The accumulation of the resveratrol in the dermis was significantly higher for glycerosomes than for liposomes and PG-PEVs (1.0% and 0.4%, respectively). Using propylene glycol or glycerol in the delivery systems increased their viscosity and in turn the better spreadability was achieved. In addition, the increased viscosity resulted in better stability of PEVs and glycerosomes compared to liposomes [135].

Incorporation of resveratrol into liposomes inhibited the cytotoxic effect of resveratrol at 100 µM concentrations on HEK 293 cells [141]. Moreover, the liposomes avoided its immediate and massive intracellular distribution and increased resveratrol’s ability to stimulate proliferation of the cells and to survive under stress conditions caused by UV-B light. Previously, it was demonstrated that topical application of resveratrol on the hairless skin of mice exerts a chemopreventive effect against damage caused by exposure to UV-B radiation via inhibiting survivin [142].

Detoni et al. [143] prepared several nanocarriers (liposomes, polymeric lipid-core nanocapsules and nanospheres, and solid lipid nanoparticles) to evaluate prevention from photodegradation of the E-isomeric form of resveratrol (E-RSV). Liposomes with saturated soy phosphatidylcholine were obtained with a standard method of thin lipid film hydration followed by high-pressure homogenization. The authors observed that liposomes had the highest polydispersity index (PI) among all prepared structures (0.29 vs. 0.12 for nanostructured lipid carriers and 0.16 for nanospheres, respectively). However, PI for all formulations obtained was below 0.3 which suggested that each preparation method led to controlled size distributions. Again, the highest photostability of the E-RSV was demonstrated in liposomes with a t1⁄2 of 592.2 ± 9.6 min. After 8 h of UVA radiation, a second population of smaller particles appeared for the E-RSV-loaded liposomes [142]. However, obtained liposomes presented a poor physical stability and according to authors, lipid-core nanocapsules and NLC were the most adequate formulations for E-RSV.

Transfersomes are one of the carriers ideal for poor soluble substances. Similarly to liposomes, they are composed of phospholipids and water with the addition of surfactant. The bilayer lipid membrane of transfersome is more flexible than the membrane of liposomes. Using transfersomes, a low and high molecular weight drug may be delivered to the skin with a great efficacy [144]. Wu et al. [145] prepared resveratrol-loaded transfersomes using a high-pressure homogenization technique. The highest accumulation was found for D1–20(W) formulation containing Tween-20 and accounted for 5.18 µg/cm^2^ at 6 h while for RSV not encapsulated—4.06 µg/cm^2^ [145]. Additionally, authors demonstrated that transfersomes decreased the cytotoxicity of resveratrol, and the viability of the cells treated with transfersomes was significantly improved.

Abbas and Kamel [146] used a thin film hydration method to prepare “spanlastics” (surfactant-based elastic vesicles) from different ratios of Span 60 (S60) and edge activators (EAs). Such a system for resveratrol delivery may be a promising approach to prevent UV-induced skin damage.

Among four types of liposomes with resveratrol, including ethosomes, ultradeformable liposomes, deformable liposomes, and conventional liposomes, the best encapsulation efficiency was observed for deformable liposomes with resveratrol (91.8%) while the least, for ultradeformable liposomes with RSV [147]. In the study by Scognamiglio et al. [148], two types of nanovesicles containing trans-resveratrol were evaluated, i.e. transfersomes and ethanol-containing vesicles. In in vitro testing the authors demonstrated that only ethanol-containing vesicles based on soy phosphatidylcholine were able to promote the permeation of trans-resveratrol through the porcine skin. As to cytotoxicity, blank nanocarriers containing Tween 80 or ethanol were cytotoxic against HaCaT cells which led to a higher level of ROS; however no cytotoxicity was observed for vesicles containing trans-resveratrol. Table 3 shows results of the studies regarding the topical usage of different types of liposomes containing resveratrol. 

#### 4.2.3. Polymeric Nanoparticles

Composite nanoparticles were prepared to enhance the solubility and dissolution properties of trans-resveratrol using a supercritical antisolvent process [149]. The composition of the prepared nanoformulations was as follows: trans-resveratrol and hydrophilic polymers hydroxylpropylmethyl cellulose (HPMC) at 1:4 and 1:5 ratios of trans-resveratrol/HPMC/surfactants (poloxamer 407, d-α-Tocopherol polyethylene glycol 1000 succinate (TPGS), and gelucire 44/14 at 1:4:1 ratio. Ex vivo skin permeation studies were carried out with Franz diffusion cells, using the skin of rats as the diffusion membrane. The oral bioavailability of trans-resveratrol composite nanoparticles was investigated in male rats. In ex vivo conditions, it was shown that among the tested formulations, the most resveratrol penetrated the skin from trans-resveratrol/HPMC/poloxamer 407 nanoparticles (1:4:1). According to the authors, PMC/surfactant nanoparticles are promising formulations enhancing the absorption of trans-resveratrol after oral administration for use in healthcare products as well as for skin application of cosmetic products.

Another research team [150] constructed zein nanoparticles (0.5% *w*/*v*) containing resveratrol (0.025% *w*/*v* grape skin extract) coated with Maillard conjugates (MC) of sodium caseinate and different molecular mass carbohydrates. Zein, as a protein-based material, is renewable, biodegradable, and relatively inexpensive compared to animal proteins (e.g., gelatin and albumin). Its disadvantage as a delivery vehicle is its low stability. The nanoparticles produced by Davidov-Pardo et al. [150] were stable against environmental stresses, (e.g., temperature, pH, and ionic strength changes) and their stability increased with increasing carbohydrate molecular weight. Conjugates had no effect on encapsulation efficiency (∼83%) however, significantly improved particle stability against changes in pH, CaCl_2_ addition, and heat treatment.

A promising material in the regulation of pigmentation of skin exposed to UVB, has been shown to be genetically modified rice, enriched with resveratrol (resveratrol-enriched rice, RR), having synergistic anti-inflammatory effects of resveratrol and normal rice [151]. Rice is added to lightening cosmetic products, and to prevent aging and wrinkle formation [152,153]. Rice ingredients improve the skin barrier function, preventing water loss from the epidermis, by regulating level lipids such as glucosylceramides and ceramides [154]. Lee et al. [155] obtained particles in the nanometer range from RR extract using high-pressure homogenization technology. The prepared micro- and nanoparticles (micro-cRR and nano-cRR, 200 μL 1% (*w*/*v*)), were applied topically to the dorsal skin of guinea pigs, previously exposed to UV-B radiation for 15 days. Based on the results, they showed that nano-RR showed a greater depigmentation effect than micro-cRR. In skin samples taken immediately after the guinea pigs were sacrificed, higher concentrations of resveratrol were determined in the nano-cRR group (about 15 times) than in the micro-cRR group. In histological examination, it was observed that melanin granules produced by UV-B overirradiation decreased by 160% from nano-cRR treatment. In turn, based on the immunohistochemical studies performed, the authors found that resveratrol secreted from nano-cRR significantly downregulated the protein level of tyrosinase, TRP-1 and TRP-2.

Silk proteins are also an interesting biomaterial whose potential is currently being explored for use in controlled drug delivery. Previously, silk proteins, in the form of hydrogels or creams, have been used with positive results in the treatment of burn wounds in regenerative medicine, in scar reduction, and the prevention of infections [156,157,158,159,160]. The lack of disease progression after 4 weeks of treatment was confirmed by the concentration of pro-inflammatory interleukins (IL-1β and TGF-1β) as well as by the following anatomopathological measurements: low level of vessel formation and smaller collagen compaction in the samples tissue gum samples. Using a solventless precipitation technique, Suktham et al. [161] obtained resveratrol-loaded sericin nanoparticles with an average size of approximately 200–400 nm, from which resveratrol was released in vitro for over 72 h. Moreover, it has been shown that they strongly inhibited the growth of colorectal adenocarcinoma (Caco-2) cells, although they demonstrated non-cytotoxicity to skin fibroblasts.

#### 4.2.4. Silver and Gold Nanoparticles

The dynamic development of nanotechnology has led to the development and application of nanomaterials on a large scale. Metallic nanoparticles, such as AuNPs and silver nanoparticles (AgNP), which serve as antibacterial agents, deserve special attention [162,163]. The use of AuNPs and AgNPs as antibacterial agents provides an advantage over conventional antibiotics because AuNPs and AgNPs use multifaceted antimicrobial mechanisms and are therefore unlikely to increase bacterial resistance [164,165,166].

Park et al. [167] synthesized carriers for resveratrol; one type of gold nanoparticle (AuNPs) and two types of silver nanoparticle (Res-AgNPs) (by adding of sodium dodecyl sulfate (SDS)—Res-AgNPs-SDS or NaOH—Res-AgNPs-NaOH). In order to assess the in vitro antibacterial activity of gold and silver nanoparticles, twenty-two strains of Gram-positive and Gram-negative strains were screened. In the antibacterial tests it was observed that among the tested strains, *Streptococcus pneumoniae* was the most sensitive strain. The antimicrobial activity of Res-AuNPs (MIC 14.25 µg/mL) against *S. pneumoniae* was observed to be twice as high as that of the resveratrol standard (MIC 28.5 µg/mL). In contrast, Res-AgNPs-SDS (MIC 40 μg/mL) and Res-AgNPs-NaOH (MIC > 80 μg/mL) did not improve the antibacterial activity against *S. pneumoniae*. The presence of sodium dodecyl sulfate in the Res-AgNPs increased antimicrobial activity (two-fold increase) against *Staphylococcus aureus* (SG511, 285 and 503), *Escherichia coli* DC 2, *Klebsiella oxytoca* 1082E, and *S. pneumoniae* compared to Res-AgNPs-NaOH.

In turn, Orlowski et al. [168] prepared bimetallic Au@AgNPs by growing an Ag layer on AuNPs and further modified with selected polyphenols: gallic acid (a building unit of tannic acid), gallic acid derivatives (epicatechin gallate, epigallocatechin gallate). An in vitro and in vivo model showed that treatment with bimetallic nanoparticles modified with different polyphenolic compounds resulted in a significant improvement in wound healing. According to the authors, the prepared formulations obtained by combining Au@AgNPs with selected polyphenols may find application in the treatment of various types of injuries such as chronic, infected, or hyperproliferative wounds. Table 4 demonstrates a summary of data on nanoformulations of resveratrol for topical application.

#### 4.2.5. Other Nanocarriers in Topical Delivery of Resveratrol

There are little data considering usage of other nanoparticles (e.g. carbon nanotubes, dendrimers) for skin delivery of resveratrol. The vast majority of these studies concern potential usage of nanoemulsion; however, studies regarding using dendrimers as well as cubic-shaped structure (cubosomes) for transdermal delivery of resveratrol dendrimers containing resveratrol were also found [169,170]. Nanoemulsions have very small droplet sizes (between 20 to 500 nm) which favors better drug absorption, and targeting is one of the advantageous dosage forms [171]. The core of the particle is either oil (*o*/*w* form) or water (*w*/*o* form). Nanoemulsions allow to increase drug loading, enhance drug solubility and bioavailability, as well as protect the active substance from enzymatic degradation [172].

The results of the study by Sharma et al. [173] demonstrated the suitability of gels with resveratrol-loaded nanoemulsion when the topical effect is required. The authors observed that resveratrol deposition in rat skin increased in the following order: conventional dispersion (22.42 ± 1.32%), conventional gel (33.42 ± 2.14%), nanoemulsion (45.65 ± 4.76%), and nanoemulsion gel (62.65 ± 4.98%). In addition, confocal analysis showed deeper penetration of resveratrol from nanoemulsion gel which enhanced fluidization of stratum corneum lipids and conformational disruption of lipid bilayer [173].

Recently, terpenes added to nanoemulsion were shown to increase the permeation of resveratrol through the skin; the permeation increased with an increasing lipophilicity of the terpene [174]. The highest penetration of resveratrol into the stratum corneum and the epidermis-dermis-follicle region was observed in the case of nanoemulsion containing eugenol. In turn, the highest permeation of resveratrol through the skin was demonstrated for the limonene-containing nanoemulsion [174]. In the study by Khurana et al. [175], the skin deposition of resveratrol from nanoemulgel was over 6.5-fold higher than from conventional carbomer gel. The obtained carbomer-based nanoemulgel with resveratrol with globule size of 168.3 nm had optimal physicochemical properties, including viscosity, spreadability, pH, and physical stability.

Three different polyphenols, i.e., curcumin, thymoquinone, and resveratrol, were analyzed in the nanoemulsion formulations containing oleic acid as oil phase, Tween 20 as surfactant, and PEG 200 as co-surfactant [176]. Drug-loaded nanoemulgel (DLNE) exhibited a higher percentage of growth inhibition in comparison to the drug solution (DS). The highest percentage of resveratrol permeated from DLNE (33.61 ± 2.19) while the lowest from DS (10.45 ± 1.67). The authors also observed anti-angiogenic effects of DLNE for all three substances [176].

Dendrimers are multivalent and monodispersed polymers showing tree-like structure, which contain three characteristic elements: a core (or focal group), the interior of the polymer and a surface (a periphery) [177]. Pentek et al. [169] obtained polyamidoamine (PAMAM) generation 4 dendrimers (PAMAM G4) with resveratrol entrapped. As for other nanoparticles enhancing resveratrol solubility, the stability and transdermal permeation can also be assumed in the dendrimeric formulation. The ionization state at particular pH of both an active substance and dendrimer is the most important factor in complexation of the drug into dendrimer [169]. The authors demonstrated increased loading and skin penetration of resveratrol. In the study, 100% dissolution of the dendrimer-resveratrol formulations was observed within 20 min in both the simulated gastric and simulated intestinal fluids while resveratrol alone needed 4.5 h. Again, the transdermal permeation of the dendrimer-resveratrol formulation was higher compared to resveratrol alone (78.06% vs. 37.33%, respectively) [169].

In turn, improved permeation and deposition of resveratrol in mice skin layers was also demonstrated in a recent study on carbopol gel with cubosomes containing resveratrol [170]. The cubosomes had a mean particle size of 113 ± 2.36 nm and the entrapment efficiency accounted for 85.07 ± 0.91% [170].

## 5. Conclusions

Despite the fact that resveratrol is a multi-potent compound which can be used in the therapy of wide spectrum of diseases including skin diseases, its properties, including low water solubility and poor stability, may cause limitations that need to be overcome for efficient therapy. Therefore, increased water solubility and enhanced stability of resveratrol are needed for any commercially successful formulation. Such an improvement can be obtained by encapsulating resveratrol into multifunctional delivery systems, e.g. in micro- and nanoparticles. One of the most important steps in preparing micro- and nanocariers formulations is the selection of an appropriate encapsulating substance which determines the mechanism and rate of API release. The usage of biodegradable polymers is obviously of particular interest since the fact of adverse health effects which may result from the use of such carriers is the great unknown, especially in the case of long-term therapy by patients.

Available data on topical formulations with resveratrol-loaded micro- and nanocarriers show potential for such nanosystems. Resveratrol-loaded nanoformulations may be considered clinical equivalents to standard treatments with synthetic antioxidants, but with minimized side effects. They allow for targeting action as well as for decreasing the dose of the substance in comparison to conventional formulations. Due to the growing problem of antibiotic resistance, the possibility of including natural substances in clinical treatment, e.g., from the group of polyphenols, including resveratrol, is of particular importance in order to prevent this phenomenon. The interests of scientists are focused especially on clinical, drug-resistant strains of microorganisms that pose a health and even life threat to patients in serious condition. The antimicrobial activity of resveratrol is widely documented in in vitro research, which opens up the possibility of using resveratrol as a treatment adjunct. In addition, the process of its encapsulation to micro- and nanocarriers would allow to reduce the dose of the antibiotic while maintaining the effectiveness of the treatment.

Another important goal of many studies is the search for new formulations with reduced toxicity, simultaneously ensuring the targeted transport of the active substance to the neoplastic tissue. Such formulations of resveratrol may also provide a synergistic effect of drugs both administered simultaneously, e.g., by oral route or co-loaded with resveratrol in the nanoparticle. Results of some studies revealed that resveratrol alone showed insufficient action. However, systematic analyses in the topic are still scarce and incomplete as they are performed mainly in vitro. More in vivo studies are needed, especially clinical trials based on large numbers of patients, to confirm the effectiveness of novel nanoformulations containing resveratrol in the management of skin diseases.

## Figures and Tables

**Table 1:** A summary of multidirectional action of resveratrol in skin diseases.

Action of Resveratrol	Outcome	Reference
Anti-microbial activity	Topical formulation with 0.001% trans-resveratrol effectively reduces the severity of acne vulgaris.	[67]
	Has anti-HSV activity, effectively reducing or inhibiting viral replication.	[71]
	Topical application of cream containing resveratrol to skin lesions in mice SKH1 infected with HSV-1 is effective in inhibiting lesions compared to the control group.	[72]
	Topical application of 15% and 30% oxyresveratrol ointment significantly reduced lesion development in HSV-1 infected mice compared with control.	[74]
Wound healing	Together with metformin, improves vascularization of the wound beds in both young and aged rats.	[81]
	A biodegradable dressing synthesized from chitosan and sodium hyaluronate with resveratrol accelerates the formation of granulation tissue, facilitating wound healing.	[83]
	The system of bacterial cellulose with resveratrol promotes both the regeneration of epidermal tissues and re-epithelialization during wound repair in damaged rat epidermis.	[84]
	Daily oral administration of an experimental diet containing 0.04% *w*/*w* resveratrol to Sod1 mice was shown to reverse the skin thinning process associated with increased oxidative damage.	[88]
	In resveratrol-treated keloid fibroblasts, the expression of type I collagen, α-smooth muscle actin, and heat shock protein 47 was shown in a dose-dependent manner.	[92]
Melanoma	Inhibits melanoma cell viability in dose-dependent manner and influences the migration and invasion counteracting melanoma progression.	[98]
	Shows synergistic effect with radiotherapy in inhibiting melanoma cells proliferation.	[99]
	In melanoma mice model, reduced cell viability and a decrease tumor volume were found compared to free resveratrol.	[101]

HSV—herpes simplex virus; SKH hairless mice.

**Table 2:** Microformulations of resveratrol for topical application.

Type of Microformulations	Encapsulation Efficiency	Diameter Range	Main Results	Potential Therapeutic Application	Reference
HA-DPPC microparticles	≥97%	From 20 to 30 μm	Resveratrol loaded HA–DPPC microparticles performed a controlled and dose-dependent release. The GSH/GSSG levels obtained by cell culture studies acted as an evidence of the antioxidant activity of Resveratrol. The produced microparticles increased cell proliferation.	To treat chronic wounds such as diabetic ulcers	Eroğlu et al. [106]
HA-DPPC microparticles	98.7%	30.2 ± 0.3 μm	In vitro: release of resveratrol from the skin matrix according to the Weibull kinetic model. In vivo: the microparticles were well dispersed in the dermal matrix from the surface to deeper layers. Resveratrol loaded HA-DPPC microparticles enhanced wound healing process in diabetic conditions.	To treat diabetic wounds	Gokce et al. [107]
HA-DPPC microparticles	NS	30.2 ± 0.3 μm	Formulation had a significant effect on tumor necrosis factor, caspase 3, and reduced/oxidized glutathione levels. Faster wound healing was observed in patients.	To treat diabetic foot ulcer	Çetinkalp et al. [108]
(1) Lipid microparticles (LMs) uncoated (2) RSV-loaded lipid microparticles coated with chitosan	NS	(1) 5.7 μm for the uncoated LMs (2) 6.1 μm for the chitosan-coated LMs	Improved penetration of resveratrol through the human stratum corneum after application of LMs coated with chitosan was observed.	To enhance the in vivo skin permeation of resveratrol	Scalia et al. [109]
Lipid microparticles (LMs)	69.7%	12.9 ± 5.3 μm	LMs with trans-resveratrol showed its highest photostability. Light-induced degradation of trans-resveratrol was significantly reduced (from 34.3% to 19.9%) by incorporation into LMs	To enhance trans-resveratrol photostability in topical formulations	Scalia et al. [112]
(1) Microparticles prepared by sonication (SLM1) (2) Microparticles prepared by ultraturrax homogenization process (SLM2)	(1) 88.26 ± 0.98 (2) 90.00 ± 1.61	(1) 0.92 ± 1.21 μm (2) 2.80 ± 1.18 μm	An in vitro study of resveratrol-loaded SLM2 showed antioxidant activity, tyrosinase inhibition, and cytotoxicity to melanoma cell lines, and promoted apoptosis. In vivo studies of SLM2 gel in C57BL mice showed a significant reduction in tumors.	For melanoma chemoprevention	Ravikumar et al. [113]
RSV and limonene- loaded PEGylated solid lipid microspheres (SLM)	84.7%	size distribution (%): 47.5%—250 μm; 28.1%—200 μm; 18.1%—180 μm; 4.51%—150 μm; 0.7%—90 μm	The stability test showed that microencapsulated resveratrol exposed to natural light was protected from photodegradation phenomena over a period of two months. Antimicrobial tests against *S. aureus* have highlighted that microspheres possess per se antimicrobial activity, which is enhanced by the presence of limonene, and they can represent an interesting bactericide vehicle for RSV administration on the skin.	To apply as a cosmeceutical product for skincare	Angellotti et al. [114]

NS—not shown.

**Table 3:** Data on the topical use of different types of liposomes with resveratrol.

Type of Liposomes	Size of the Vesicles	Encapsulation Efficiency	Main Results	Toxicity	Reference
(1) Zwitterionic liposomes (2) Cationic liposomes	(1) From 360 ± 20 nm to 370 ± 30 nm depending on lipid ratio (2) From 110 ± 10 nm to 110 ± 15 depending on lipid ratio	NS	Cationic liposomes were more deeply inserted than zwitterionic liposomes.	Tested on stabilized cell lines (mouse fibroblast NIH-3T3 and human astrocytes U373-MG) —viability was not affected by the liposomal resveratrol.	Bonechi et al. [134]
Ultradeformable liposomes (UDL)	126.85 ± 5.30 nm	83.21 ± 1.98%	Significant stimulation of melanin and tyrosinase activity and potential antioxidant activity with a major contribution of psolaren for the former and key role of resveratrol for the latter activity.	74.95 ± 11.61% cytotoxicity at 50 μM concentration of resveratrol.	Doppalapudi et al. [52]
(1) Conventional liposomes (2) Penetration enhancer-containing vesicles (PEVs) (3) Glycerosomes	(1) 63 ± 5 nm (2) 169 ± 15 nm (3) 75 ± 5 nm	(1) 60 ± 9% (2) 70 ± 6% (3) 40 ± 4%	Improved dual activity, antioxidant and antimicrobial, were observed in the case of resveratrol and gallic acid are co-loaded in liposomes.	Assessed in HaCaT and 3T3 cells; the viability of HaCaT cells was always 92%, and even higher (115–122%) when the co-loaded resveratrol and gallic acid (2µg/mL each) were delivered by PEVs and glycerosomes.	Vitonyte et al. [135]
Liposomes	189.4 ± 14.1 nm	99.1%	The liposomes with E- RSV showed its highest photostability; liposomes presented a poor physical stability, resulting in a bimodal size distribution profile.	NS	Detoni et al. [143]
Transfersomes	From 40.13 ± 0.63 nm to 64.28 ± 0.60 nm depending on the type of edge activator	From 56.13 ± 1.52% to 59.93 ± 0.99%	Resveratrol was demonstrated to penetrate the skin more easily after encapsulation. In transdermal delivery analysis, the formulation containing Tween-20 showed increased accumulation (by 27.59%) after 6 h. All the transfersome formulations showed better accumulative penetration than unencapsulated resveratrol.	The cell viabilities of the transfersomes were all higher than 83%, among which the formulation containing Tween-80 had the lowest cytotoxicity. When the resveratrol concentrations of transfersomes were lower than 40 μM, almost no cytotoxicity was observed.	Wu et al. [145]
Spanlastics (surfactant-based elastic vesicles)	From 201.30 ± 2.45 nm to 464.80 ± 3.11 nm depending on the type of edge activator (EA) and the Span60:EA ratio	From 63.60 ± 2.89% to 79.10 ± 5.56%	Resveratrol-loaded elastic nanovesicles was demonstrated to be a promising approach to prevent UV-induced skin damage for overcoming the low drug solubility.	NS	Abbas and Kamel [146]
(1) Ethosomes (2) Ultradeformable liposomes (3) Deformable liposomes (4) Conventional liposomes	(1) 289.6 ± 0.9 nm (2) 201.3 ± 0.8 nm (3) 84.1 ± 1.0 nm (4) 220.5 ± 2.2 nm	(1) 51.0% (2) 12.6% (3) 91.8% (4) 23.0%	The increase in the fluidity of the bilayers in the region of the hydrophobic chains of the phospholipid by ethanol probably facilitates the accommodation of the resveratrol in the bilayer and contributes to the improved encapsulation of RSV without affecting the mobility of this carrier.	NS	Tosato et al. [147]
(1) Transfersomes with different surfactants (2) Ethanol-containing vesicles with different lipid composition	between 83 and 116 nm	≥70%	Only ethanol-containing vesicles prepared using soy phosphatidylcholine were able to promote trans-resveratrol permeation through the skin.	No cytotoxic effect in human keratinocytes (HaCaT) was observed in the case of nanocarriers with trans-resveratrol encapsulated.	Scognamiglio et al. [148]

NS—not shown.

**Table 4:** Summary of data on nanoformulations of resveratrol for topical application.

Type of Nanoformulations	Encapsulation Efficiency	Diameter Range	Main Results	Potential Therapeutic Application	Reference
(1) Nanoemulsions (NEs) (2) Solid lipid nanoparticles (SLNs) (3) Nanostructured lipid carriers (NLCs)	NS	(1) 27.08 ± 1.36 nm (2) from 36.40 ± 1.26 to 45.99 ± 1.30 nm (3) 26.14 ± 1.04 nm	A linear relationship between the occlusive effect in vitro and skin hydration in vivo was observed. In vivo increase in skin hydration was demonstrated for all lipid carriers, in the order SLNs > NLCs > NE.	Topical formulations with improved effectiveness	Montenegro et al. [117]
Solid lipid nanoparticles (SLNs)	68–89%	Less than 100 nm	Skin irritation test of the RES-SLN gel formulation, performed on rabbits, exhibited no irritation. Increased retention of resveratrol for SLNs through human cadaver skin was demonstrated. RES-SLN gel inhibited ear swelling and decreased water content in the skin in the BALB/c mouse model of ICD.	To treat irritant contact dermatitis	Shrotriya et al. [51]
(1) Chitosan/gum Arabic (CH/GA) nanoparticles prepared with 0.5% *w*/*v* CH/GA (2) Chitosan/gum Arabic (CH/GA) nanoparticles prepared with 1.5% *w*/*v* CH/GA	(1) 92.81% ± 1.76 (2) 94.50% ± 0.23	(1) 23.98 μm (2) 17.11 μm	Pickering emulsions stabilized by chitosan/gum Arabic (CH/GA) nanoparticles enhanced deposition of resveratrol to deeper layers skin. An ex vivo study of formulation showed higher cutaneous retention and lower permeation of resveratrol, in comparison with a control solution based on a 20% *v*/*v* ethanol.	For cosmetic applications	Sharkawy et al. [118]
(1) Microparticles-cRR (2) Nanoparticles-cRR	NS	(1) 1625.3 ± 172.5 nm (2) 1625.3 ± 172.5 nm	Resveratrol content in UVB-irradiated dermal skin tissue was a 15-fold higher after treatment with nano-cRR than that treated with micro-cRR. Nano-cRR formulation demonstrated stronger anti-melanogenic potential than micro-cRR.	To develop anti-melanogenic agents	Lee et al. [155]
(1) Solid lipid nanoparticles (2) Solid lipid nanoparticles with soy lecithin	NS	(1) 155.50 ± 0.26 nm (2) 166.23 ± 0.94 nm	The resveratrol-loaded SLNs were non-toxic to HaCat keratinocytes. The cumulative amount of resveratrol permeated through pig skin after 24 h was 45.26% for SLNs and 18.61 to SLNs with soy lecithin.	To treat skin disorders such as aging and hyperpigmentation	Rigon et al. [122]
Lipid nanoparticles. Actives: (1)Resveratrol (2) Vitamin E VE (3) Vitamin E VE acetate (4) Epigallocatechin Gallate (EGCG)	(1) 99% (2) 99% (3) 94% (4) 99%	(1) From 102 to 311 nm (2) From 86 to 169 nm (3) From 125 to 155 nm (4) From 126 to 167 nm	Lipid nanoparticles significantly improved the photostability of resveratrol compared to the control sample which was resveratrol solution in mixed ethanol and penetration of active through the stratum corneum.	For skin care applications to provide the skin with long-lasting antioxidant benefits	Chen et al. [127]
(1) HPMC/surfactant nanoparticles (2) Hydroxylpropylmethyl cellulose/surfactant nanoparticles	Over 97%	Less than 300 nm	Ex vivo skin permeation study showed the steady state flux (Jss) of trans-resveratrol from nanoparticles containing poloxamer 407 and TPGS was much higher than that of nanoparticles containing gelucire 44/14, with approximately 2.2- and 1.9-fold increases, respectively, compared to micronized trans-resveratrol.	For healthcare products (owing to their enhanced absorption via oral administration) and for skin application with cosmetic products	Ha et al. [149]
Zein particles loaded prepared with Maillard conjugates of sodium caseinate and dextran	∼83%	Less than 200 nm	Improvement of the stability of zein nanoparticles with resveratrol has been demonstrated by coating them with Maillard conjugates (MC) made from sodium caseinate and neutral carbohydrates. The molecular mass of the carbohydrates used to form the MC had a significant impact on particle stabilization, with the stability of the delivery systems increasing with increasing carbohydrate molecular mass.	Zein nanoparticles coated by MC may be delivery systems for hydrophobic bioactive molecules in a wide range of commercial products	Davidov-Pardo et al. [150]
(1) Gold nanoparticles (AuNPs) (2) Silver nanoparticles (AgNPs)—SDS (3) Silver nanoparticles (AgNPs)—NaOH	NS	From 8.32 to 21.84 nm	AuNPs and Res-AgNPs showed higher antimicrobial activity compared to resveratrol alone. The addition of sodium dodecyl sulfate during the synthesis of Res-AgNPs slightly increased their antibacterial activity.	Applications of nanoparticles as antibacterial reagents	Park et al. [167]
Bimetallic gold-silver nanoparticles modified with different polyphenol	NS	From 33 to 63 nm	Modification of bimetallic Au@AgNPs with selected polyphenols resulted in significant improved wound healing both in vitro and in vivo. The different polyphenols used to modify the bimetallic nanoparticles showed different effects at different stages of wound healing.	To wound healing	Orlowski et al. [168]

NS—not shown.

## Data Availability

Not Applicable.
